# Prevention of Bone Resorption by HA/β-TCP + Collagen Composite after Tooth Extraction: A Case Series

**DOI:** 10.3390/ijerph16234616

**Published:** 2019-11-21

**Authors:** Hsi Kuei Lin, Yu Hwa Pan, Eisner Salamanca, Yu Te Lin, Wei Jen Chang

**Affiliations:** 1 School of Dentistry, College of Oral Medicine, Taipei Medical University, Taipei 110 Taiwan; linhsikuei38@gmail.com (H.K.L.); shalom.dc@msa.hinet.net (Y.H.P.); d204103004@tmu.edu.tw (E.S.); 2 Dental Department, Taipei Medical University, Shuang-Ho hospital, Taipei 235, Taiwan; 3 Department of General Dentistry, Chang Gung Memorial Hospital, Taipei 105, Taiwan; 4 Graduate Institute of Dental & Craniofacial Science, Chang Gung University, Taoyuan 333, Taiwan; 5 Sunmax Biotech Co. Ltd., Tainan 744, Taiwan; lud32@msn.com

**Keywords:** HA/β-TCP + collagen composite, periodontal bone loss, dental sockets preservation, tooth extraction

## Abstract

After tooth extraction, alveolar ridge loss due to resorption is almost inevitable. Most of this bone loss occurs during the first six months after the extraction procedure. Many studies have indicated that applying socket-filling biomaterials after extraction can effectively reduce the resorption rate of the alveolar ridge. The purpose of this study was to investigate the clinical efficacy of the application of a hydroxyapatite/β-tricalcium plus collagen (HA/β-TCP + collagen) dental bone graft in dental sockets immediately after tooth extraction, so as to prevent socket resorption. The study was conducted on 57 extraction sockets located in the mandible and maxilla posterior regions in 51 patients. HA/β-TCP + collagen was inserted into all of the dental sockets immediately after extraction, and was covered with a flap. Follow-up was performed for three months after extraction, using radiographs and stents for the vertical and horizontal alveolar ridge measurements. A minimal alveolar bone width reduction of 1.03 ± 2.43 mm (*p* < 0.05) was observed. The height reduction showed a slight decrease to 0.62 ± 1.46 mm (*p* < 0.05). Radiographically, the bone height was maintained after three months, indicating a good HA/β-TCP + collagen graft performance in preserving alveolar bone. In conclusion, the HA/β-TCP + collagen graft demonstrated adequate safety and efficacy in dental socket preservation following tooth extraction.

## 1. Introduction

Bone resorption has long been an inevitable result of tooth extraction. Observable deformity and resorption often follow the procedure in the form of alveolar ridge reduction, both vertically and horizontally. The severity of bone loss directly affects the success rate of subsequent dental implant procedures, and the post-treatment aesthetic results [1,2,3,4,5]. Numerous studies have shown that after tooth extraction, approximately 30% of the alveolar ridge is lost as a result of resorption. Studies have shown that during the first three months after extraction, approximately two-thirds of the affected hard and soft tissues undergo some degree of resorption. Most of the bone loss occurs during the first six months after the procedure. Afterward, the resorption rate increases at a pace of 0.5–1% on average annually [3,6,7]. Moreover, an estimated 50% of the alveolar bone width is lost within 12 months after the extraction, 30% of which occurs within the first 12 weeks. Other studies have indicated that the alveolar ridge resorption is more severe on the buccal side than on the lingual side. After extraction, the lateral walls of the extraction socket undergo intense resorption, which causes a significant alveolar ridge height reduction [2,3,8,9]. On average, the height reduction is approximately 2 mm greater on the buccal side than on the lingual side [8].

In an effort to maintain adequate alveolar bone after tooth removal, and to minimize resorption, many researchers have examined the efficacy of different biomaterials in preserving extraction sockets. The use of graft materials in extraction sockets to slow socket wall resorption has already become common clinical practice. Material selection affects the success of preserving dental sockets. Other than being adequately biocompatible and easily maneuvered, an ideal dental bone graft material should possess one or more of the following characteristics: (1) osteoconductivity, to provide scaffolds that guide angiogenesis and osteogenesis; (2) osteoinductivity, to induce mesenchymal stem cells near the implant site to differentiate into preosteoblasts; and (3) osteogenicity, to contain osteoblasts that readily differentiate into new bone cells after implantation [10].

Bone graft materials can be further categorized according to their origins, as autografts, allografts, xenografts, and alloplasts [11]. An autograft is a bone graft taken from another part of the same person, and is considered the “gold standard” material. Drawbacks include the need for additional surgery and the limited availability of the autograft material. Other materials, such as xenografts and artificial bone graft materials, have been developed as options for procedures such as socket preservation. There are many kinds of artificial bone grafts; bioceramics are the materials mainly used. Biomaterials such as hydroxyapatite (HA), β-tricalcium (β-TCP), and biphasic calcium phosphate have been studied extensively, because they have inorganic constituents (Ca^2+^ and P^3+^) like those in human bones. HA, β-TCP, and their composite grafts have been studied extensively in dental and orthopedics research [12].

HA/β-TCP and type I collagen composites are made with several different ratios of HA/β-TCP and type I collagen. Both HA and collagen I are biocompatible, osteoinductive materials that make up most of the bone matrix. The two can be combined to speed up the process of osteogenesis. Collagen, the major component that constitutes the organic portion of the bone, consists of the extracellular matrix secreted by osteoblasts during osteogenesis. Collagen serves not only as the scaffold on which calcium salts deposit, but also as the model for the ossification of bone matrices. Furthermore, collagen also promotes cell migration, adhesion, and differentiation. When collagen is degraded in vivo, large amounts of amino acids are released into its surroundings; they serve as nutrients in the osteogenesis that follows. The composite of HA/β-TCP plus collagen can strengthen the mechanical integrity of the composite bone graft material, while keeping the best qualities of both materials—the malleability and plasticity of collagen I make up for the brittleness of HA. Moreover, the powder-formed collagen can also serve as an excipient—its adhesive properties can overcome the clinical shortcomings of the HA particles. In a previous study, the HA/β-TCP plus collagen composite exhibited good biocompatibility and physical properties. New bone formation was also demonstrated in an animal study [11,13]. Many clinical studies have indicated that the application of a bone graft material in extraction sockets can noticeably reduce postoperative alveolar bone resorption, both vertically and horizontally [11,13,14]. The purpose of this study was to investigate the clinical efficacy of an HA/β-TCP plus collagen dental bone graft (HA/β-TCP + collagen) in preventing bone resorption when applied to dental sockets immediately after tooth extraction.

## 2. Materials and Methods

The study was conducted over a nine-month period at the Shuang Ho Hospital Dental Department, Taipei Medical University, in 51 patients with 57 extraction sockets in the mandible and maxilla posterior region. The study was approved by the Taipei Medical University Joint Institutional Review Board (approval no. 201202005). Patients were selected according to the following inclusion criteria:

- Age between 20 and 89 years.
- Absence of systemic diseases.
- Sound structure of the extraction socket.
- Untreatable tooth that had to be extracted.
- Presence of one or more neighboring teeth near the extraction socket.

The following exclusion criteria were used to disqualify patients:

- Inability to maintain good oral hygiene.
- Presence of systemic diseases, such as immune diseases and infectious diseases.
- Treatment with radiotherapy or other types of cancer treatments during the study or six months prior.
- Presence of uncontrolled diabetes.
- Betel nut chewing.
- Pregnancy, breastfeeding, or plans to have a baby during the study.
- Anticoagulant therapy.
- Defective buccal/lingual walls.

### 2.1. Tooth Extraction and Socket Preservation Procedure

Before surgery, a periodontal probe and a stent with guides were fabricated to measure the changes in the alveolar ridge during the study period. The measurements were first performed on the day of surgery and then preoperatively by a trained dental professional, who also performed the posterior follow-up measurements.

**Figure 1:**
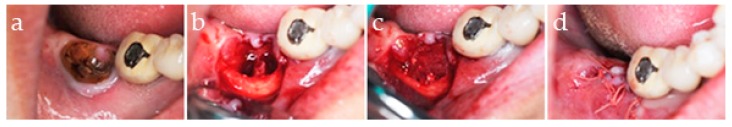
Surgical socket preservation with composite of hydroxyapatite/β-tricalcium plus collagen (HA/β-TCP + collagen). (**a**) Preoperative appearance. (**b**) Appearance immediately after extraction. (**c**) Socket filled with an HA/β-TCP + collagen composite. (**d**) Appearance after suturing: complete socket closure.

A trained periodontist performed all of the surgeries, following the same protocol. Local anesthetic (2% lidocaine and 1.7 mL Xylestesin-A; 3M ESPE, Neuss, Germany) was injected into the extraction zone. Later, a full flap was created to cover the mesial and distal vertical heights of the socket, and then the tooth was gently removed with minimal trauma to the surrounding tissue. Curettage of the socket was performed to remove any granulation tissue. After this, the HA/β-TCP + collagen composite (collagen dental bone graft, Sunmax Biotechnology, Tainan, Taiwan) was packed into the extraction socket, until its level reached 1 mm above the buccal plate (Figure 1c). The unoccupied spaces were later covered by the full flap with simple sutures (polyglycolic acid, absorbable EU-TEK, and VC194L suture; Chia-Ho Tech Co Ltd., Taipei, Taiwan; Figure 1).

The material used was a plug of thoroughly blended purified porcine type I collagen and HA/β-TCP, with a weight ratio of 30:70, respectively. In the HA/β-TCP used in the mixture, the ratio of hydroxyapatite to β-tricalcium phosphate was 60:40. The mixture was poured into a plug mold and freeze-dried [11,13].

**Figure 2:**
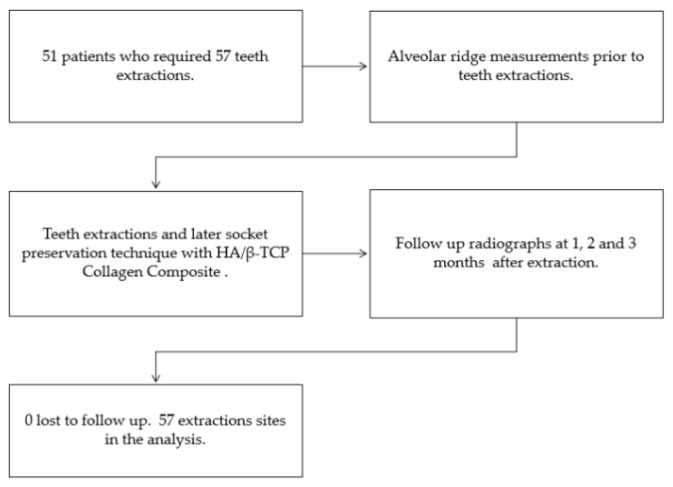
Socket preservation with a composite of hydroxyapatite/β-tricalcium (HA/β-TCP) + collagen: flow diagram of the study.

The graft material and the flaps were left to heal, without mechanical or tension stress (Figure 2). Each patient received post-surgical instructions about care, and was prescribed amoxicillin with clavulanic acid (Augmentin; SmithKline Beecham Plc, Middlesex, London, United Kingdom), 375 mg three times a day, for antibiotic prophylaxis, and acetaminophen (Panadol; GlaxoSmithKline, Dungarvan, Ireland), 500 mg three times a day, for pain control. Follow-up visits were conducted every month for three months (Figure 2).

### 2.2. Measurements by Stent with Guides before Extractions and during Follow-Up

#### 2.2.1. Changes in Alveolar Ridge Width

As mentioned, the alveolar ridge width was measured with a periodontal probe from the buccal to palatal/lingual (Qulix Periodontal Probe CP-2 Single End #30 Standard Ea, Hu-Friedy) through the middle of the socket, by determining the distance between the adjacent teeth. The same periodontal probe was used to measure the level of alveolar bone width one, two, and three months after the extraction. The width preservation level was calculated as 1 – (preoperative width baseline value − width measurements [1, 2, or 3 m after extraction])/preoperative baseline width.

#### 2.2.2. Changes in Alveolar Ridge Height

**Figure 3:**
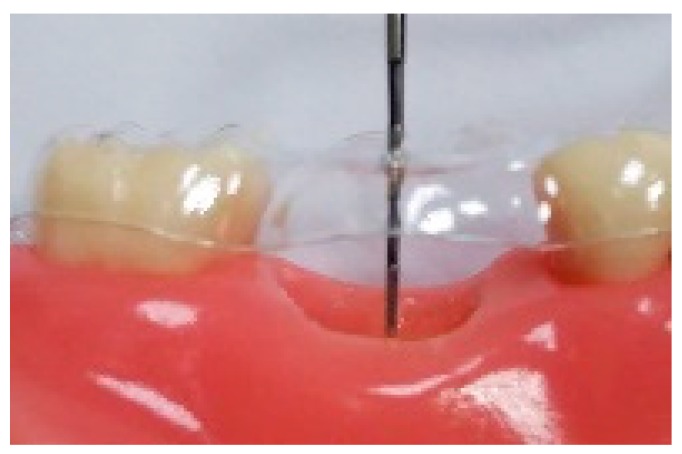
Changes in the alveolar ridge. Alveolar ridge height measurement.

The alveolar ridge height was calculated from an acrylic resin surgical stent with a ledge and vertical grooves, made for each patient. The vertical grooves were used as a point of reference for the periodontal probe, which was used to measure the alveolar ridge height from the stent to the highest level of the dental socket, including the soft tissue (Figure 3). The preservation level of the alveolar bone height was measured one, two, and three months after the extraction. The height preservation level was calculated as 1 − (preoperative height baseline value − height measurement [1, 2, or 3 m after extraction])/preoperative baseline height.

#### 2.2.3. Radiographic Measurement

The alveolar bone height was measured preoperatively, as well as one, two, and three months after the surgery, with digital periapical radiographs by an X-ray technician. A horizontal line connecting the cementoenamel junction of the teeth next to the surgical area was drawn and used as a reference to compare the one-, two-, and three-month radiographs, and to measure from the same reference line. We used professional image recombination software (EZ-Dental; Asahi Roentgen Co Ltd., Kyoto, Japan). The height preservation mean level was calculated as 1 − (preoperative height baseline value − height measurement [1, 2, or 3 months after extraction])/preoperative baseline width.

### 2.3. Statistical Analysis

One-way analysis of variance with post hoc testing was used to compare the differences in the alveolar ridge width and height, and in the radiographic height measurements from month one to month three in each group. The results were considered statistically significant if *p* was less than 0.05.

## 3. Results

We studied a total of 57 surgical sites in 51 patients, of which 28 were male and 23 were female. The oldest patient in the study was 73 years of age and the youngest was 27; the average age was 50 years old. Most of the patients were between the ages of 41 and 60 years old (Table 1).

### 3.1. Changes in Alveolar Ridge Width

**Figure 4:**
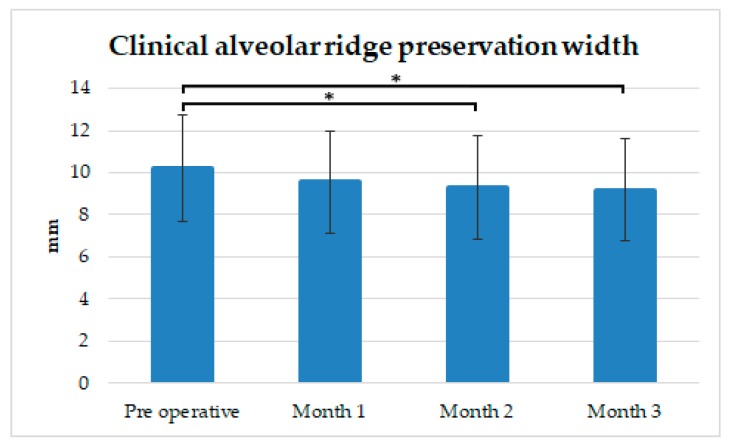
Alveolar ridge width preservation. The efficacy of the composite of HA/β-TCP + collagen in preserving the alveolar bone width was good. * Significant difference between preoperative and later measurements: *p* < 0.05.

As shown in Figure 4, and Table 2 and Table 3, the average alveolar ridge width preoperatively was 10.21 ± 2.5 mm; this measurement served as the reference point for later measurements. One month after extraction, the average alveolar bone width showed an average loss of 0.66 ± 2.42 mm; two months after extraction, the alveolar ridge exhibited an average width loss of 0.89 ± 2.45 mm (*p* < 0.05); and three months after extraction, a loss of only 1.03 ± 2.43 mm was observed (*p* < 0.05). After three months, 32 alveolar bone sites exhibited a 1-mm bone loss; 12 sites, a 2-mm loss; 9 sites, between 0- and 2-mm gains; and only 1 site, a 3-mm loss.

### 3.2. Changes in Alveolar Ridge Height

**Figure 5:**
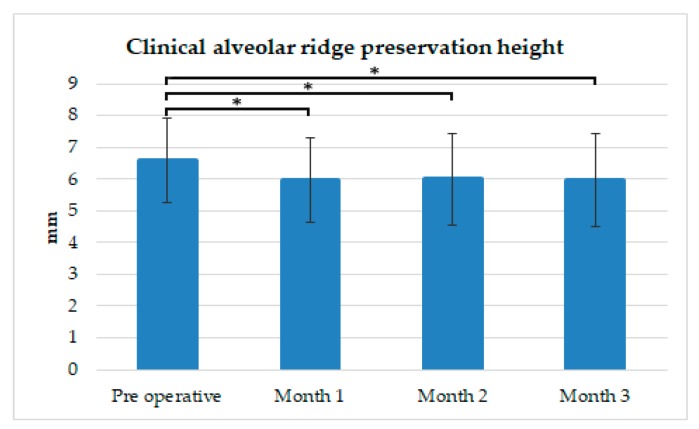
Alveolar ridge height preservation. The composite hydroxyapatite/β-tricalcium (HA/β-TCP) + collagen for alveolar bone height preservation was always slightly more efficacious after the preoperative measurement. * Statistically significant difference between preoperative and postoperative measurements: *p* < 0.05.

As shown in Figure 5, and Table 4 and Table 5, the average vertical measurement from the stent to the alveolar ridge was 6.57 ± 1.32 mm; this measurement served as the reference point for later measurements. One month after extraction, the alveolar bone height decreased by an average of −0.61 ± 1.33 mm (*p* < 0.05). Two months after extraction, the average alveolar bone height was almost the same as that in the previous month, losing an average of only 0.57 ± 1.44 mm (*p* < 0.05). Three months after extraction, the average loss in alveolar bone height was 0.62 ± 1.46 mm (*p* < 0.05). After three months, 15 alveolar bone sites exhibited a 1-mm bone loss; 13 sites demonstrated no loss; 12 sites showed a 0.5-mm loss; and 11 sites between a 1.5- and 2-mm loss. In contrast, seven sites showed a gain of 1 mm.

### 3.3. Radiographic Measurement

**Figure 6:**
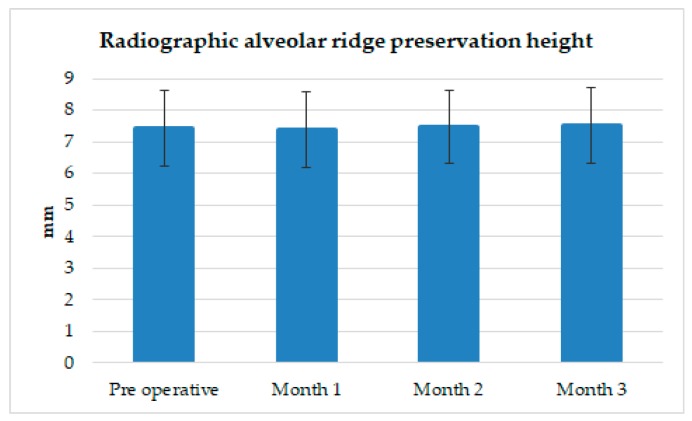
Radiographic measurement of the alveolar ridge height preservation. HA/β-TCP + collagen is efficacious in preserving the alveolar bone height, with no statistically significant difference between the postoperative measurements and the preoperative measurement (*p* > 0.05).

**Figure 7:**
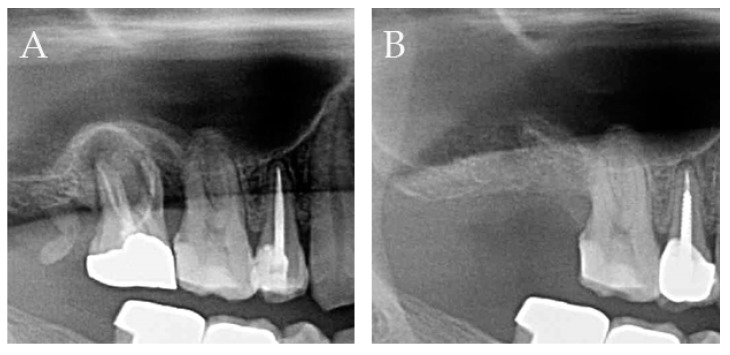
Alveolar ridge height preservation. (**A**) Preoperative. (**B**) Three months after extraction.

As shown in Figure 6 and Figure 7, and Table 6, the bone height stayed almost the same over three months. At the preoperative measurement, the distance between the imaginary cementoenamel line and the alveolar ridge was 6.62 ± 1.32 mm, which served as the point of reference for the subsequent measurements. One month after extraction, the alveolar bone height decreased almost imperceptibly (0.02 ± 1.20 mm). Two months after extraction, the alveolar bone height increased only 0.05 ± 1.14 mm. Three months after extraction, the alveolar bone height increased 0.11 ± 1.20 mm. According to the statistical analysis, the alveolar ridge height showed no statistically significant difference on the radiographs between the preoperative measurement and the three subsequent measurements.

## 4. Discussion

The purpose of this study was to investigate the clinical efficacy of the composite of HA/β-TCP + collagen as a dental bone graft, to prevent bone resorption when applied to dental sockets immediately after tooth extraction. HA/β-TCP + collagen demonstrated a good efficacy in preserving the alveolar bone width and height through osteoconduction in patients with thin biotypes. Through the histological evaluation of the horizontal alveolar ridge augmentation with β-TCP, Shalash et al. demonstrated that this material has adequate osteoconductive properties [15]. Their results indicated that patients with surgical sites filled with an HA/β-TCP + collagen graft experienced common adverse postoperative symptoms, such as pain and local inflammation, usually in association with the dental extraction rather than with the graft. Most of these adverse reactions occurred within 10 days after the procedure; all of the symptoms mentioned leveled off without any complication. Other adverse effects, such as headaches and common cold symptoms, were deemed unrelated to the product. Hong et al. found that after a two-week healing period, all of the experimental sites were fully closed with the gingival epithelium [16]. Of the greatest importance is that no adverse tissue reaction, infection, or delayed healing occurred.

The healing of extraction sockets is a highly dynamic process; a cascade of inflammatory responses is activated immediately upon tooth removal. The healing process takes approximately 12 to 16 weeks. According to Schropp et al., about two-thirds of the affected hard and soft tissues undergo some degree of resorption change during the first three months after the extraction [3]. As mentioned, approximately 50% of the alveolar bone width is lost within 12 months after the extraction, and 30% (a 3.8-mm change) occurs within the first 12 weeks, mainly because of the loss of the buccal plate of the alveolar bone. Therefore, techniques for preserving the extraction socket have been heavily researched [17,18,19,20]. In our study, HA/β-TCP + collagen demonstrated a statistically significant efficacy for maintaining the alveolar ridge height after three months, according to the periapical radiographs; a loss in height of 0.62 ± 1.46 mm, and a loss in width of 1.03 ± 2.43 mm indicated more of a reduction in soft tissue, which reflected the patients’ thin biotypes.

The use of an HA/β-TCP + collagen graft in this study may have reduced or eliminated the need for future alveolar ridge augmentation. Further studies with longer follow-up periods are necessary for determining whether the three-month follow-up results can be maintained. Such an outcome would indicate the material’s reliability in minimizing dental socket-related wall resorption. Araújo et al. also demonstrated that the grafting of osteoconductive biomaterials, such as HA/β-TCP + collagen, could reduce bone resorption and preserve the dental socket after extraction, and so it could be used for later treatments, such as dental implant placement [9,21,22,23,24]. In a meta-analysis, Avila-Ortiz et al. determined that flap elevation, the use of a membrane, and the application of a xenograft or an allograft are associated with superior outcomes, particularly with regard to the preservation of mid-buccal and mid-lingual height [17].

Extraction socket preservation helps prevent and reduce the rate of resorption of alveolar ridge, and the subsequent recession of interdental papilla, and thus helps preserve an ideal bone height, width, and density for later implant placement. This technique also helps restore the interdental papilla to a normal height for aesthetic purposes. In previous studies, investigators have mentioned several important steps for preserving the alveolar ridge, such as noninvasive extraction, closure of the extracted tooth cavity, and filling the dental socket to induce osteogenic processes [17]. We followed all these steps, and even though we did not conduct a histological analysis to demonstrate the rate of material resorption and new bone formation inside the socket, the periapical radiograph images indicated that we avoided potential errors caused by the interference of the surrounding soft tissue during probing; they also demonstrated that inside the sockets, the radiopaque trabeculae resembled the patient’s bone. The discrepancy between the changes in the clinical height and the radiographic measurements can be explained by the thinness of the gingiva tissue covering the surgical area.

As mentioned, research has been dedicated to finding osteogenic materials that can fill the extraction socket, in the hopes of retaining the bone walls around the dental socket and minimizing the resorption process that soon follows. In our study, the composite bone graft material being tested was made of 60% hydroxyapatite and 40% calcium phosphate, and the material was further homogenized with collagen to aid in preserving the alveolar ridge and to make the bovine bone graft more manageable. During the first 24 h after extraction, the most important step in socket healing is the formation of a blood clot. While blood clots are formed, platelets release growth factors, such as tumor necrosis factor α and platelet-derived growth factor, to promote the differentiation of new cells. During the next 48 h, the advent of angiogenesis causes more undifferentiated cells from other parts of the body to take part in the healing process. Under such physiological conditions, the unique porous feature of an HA/β-TCP + collagen graft material can serve as the scaffold for blood clot attachment, and the subsequent angiogenesis. The inorganic materials in the bone graft provide adequate strength to support the bone plates, thereby preventing alveolar ridge resorption.

The slow rate of resorption of some biomaterials, such as HA/β-TCP, could be considered a clinical advantage because the alveolar ridge contour can stabilize [25]. In addition, past studies have shown that β-TCP may be involved in the process of healing, such as the formation of blood clots, replacement of granulation tissue with bone precursor matrix, and formation of reticular bone. β-TCP is initially adsorbed, later replaced by interstitial tissue, and eventually mineralized to form new bone tissue [9,23,26,27]. Collagen was proposed for socket preservation so as to protect the HA/β-TCP particles, induce blood clot formation, and stabilize the wound [28,29]. Because the material is a hemostatic agent, it can stimulate platelet aggregation and enhance fibrin linkage, which may lead to initial clot formation, stability, and maturation. Furthermore, the chemotactic properties of collagen could enhance cell migration and promote primary wound coverage, which are fundamental for bone growth [20].

## 5. Conclusions

Despite the small sample size in this study, the use of an HA/β-TCP + collagen dental bone graft in the postoperative preservation of the alveolar ridge was shown to be feasible, and it can be considered biocompatible and safe as well, inasmuch as only common postoperative adverse reactions were observed. In terms of efficacy, the HA/β-TCP + collagen dental bone graft can be considered an option for preserving alveolar bone height.

## Figures and Tables

**Table 1:** Study demographics.

Sex	Male (*n*)	23
	Female (*n*)	28
Age (years)	Mean ± SD	50.40 ± 11.48
	Range	27 to 73
Total sites treated		57

SD—standard deviation.

**Table 2:** Variation in alveolar width variation by time of measurement.

Time of Measurement	Average Change in Alveolar Width (mm)
Preoperative	0
One month after extraction	−0.66 ± 2.42
Two months after extraction	−0.89 ± 2.45
Three months after extraction	−1.03 ± 2.43

**Table 3:** Differences in alveolar site widths three months after extraction.

Variation (mm)	2	1	0	−1	−2	−3
Number of alveolar sites	1	4	4	32	12	3

**Table 4:** Variation in alveolar height variation by time of measurement.

Time of Measurement	Average Change in Alveolar Height (mm)
Preoperative	0 ± 1.3
One month after extraction	−0.61 ± 1.33
Two months after extraction	−0.57 ± 1.44
Three months after extraction	−0.62 ± 1.46

**Table 5:** Change in alveolar sites heights three months after extraction.

Variation (mm)	−2	−1.5	−1	−0.5	0	1
Number of alveolar sites	6	5	15	12	13	7

**Table 6:** Variation in radiographic alveolar height by time of measurement.

Time of Measurement	Average Radiographic Change in Alveolar Bone Height (mm)
Preoperative	0
One month after extraction	−0.02 ± 1.20
Two months after extraction	0.05 ± 1.14
Three months after extraction	0.11 ± 1.20
